# Argon plasma improves the tissue integration and angiogenesis of subcutaneous implants by modifying surface chemistry and topography

**DOI:** 10.2147/IJN.S167637

**Published:** 2018-10-08

**Authors:** Michelle Griffin, Robert Palgrave, Víctor G Baldovino-Medrano, Peter E Butler, Deepak M Kalaskar

**Affiliations:** 1 UCL Centre for Nanotechnology and Regenerative Medicine, Division of Surgery and Interventional Science, University College London, London, UK, d.kalaskar@ucl.ac.uk; 2 Royal Free London NHS Foundation Trust Hospital, London, UK; 3 The Charles Wolfson Center for Reconstructive Surgery, Royal Free London NHS Foundation Trust Hospital, London, UK; 4 Department of Chemistry, University College London, London, UK; 5 Laboratory of Surface Science (SurfLab), School of Chemical Engineering, Piedecuesta, Colombia; 6 UCL Institute of Orthopaedics and Musculoskeletal Science, Division of Surgery and Interventional Science, University College London, London, UK, d.kalaskar@ucl.ac.uk

**Keywords:** tissue integration, angiogenesis, surface modification, biomaterials, implants

## Abstract

**Background:**

Tissue integration and vessel formation are important criteria for the successful implantation of synthetic biomaterials for subcutaneous implantation.

**Objective:**

We report the optimization of plasma surface modification (PSM) using argon (Ar), oxygen (O_2_) and nitrogen (N_2_) gases of a polyurethane polymer to enhance tissue integration and angiogenesis.

**Methods:**

The scaffold’s bulk and surface characteristics were compared before and after PSM with either Ar, O_2_ and N_2_. The viability and adhesion of human dermal fibroblasts (HDFs) on the modified scaffolds were compared. The formation of extracellular matrix by the HDFs on the modified scaffolds was evaluated. Scaffolds were subcutaneously implanted in a mouse model for 3 months to analyze tissue integration, angiogenesis and capsule formation.

**Results:**

Surface analysis demonstrated that interfacial modification (chemistry, topography and wettability) achieved by PSM is unique and varies according to the gas used. O_2_ plasma led to extensive changes in interfacial properties, whereas Ar treatment caused moderate changes. N_2_ plasma caused the least effect on surface chemistry of the polymer. PSM-treated scaffolds significantly (*P*<0.05) enhanced HDF activity and growth over 21 days. Among all three gases, Ar modification showed the highest protein adsorption. Ar-modified scaffolds also showed a significant upregulation of adhesion-related proteins (vinculin, focal adhesion kinase, talin and paxillin; *P*<0.05) and extracellular matrix marker genes (collagen type I, fibronectin, laminin and elastin) and deposition of associated proteins by the HDFs. Subcutaneous implantation after 3 months demonstrated the highest tissue integration and angiogenesis and the lowest capsule formation on Ar-modified scaffolds compared with O_2_- and N_2_-modified scaffolds.

**Conclusion:**

PSM using Ar is a cost-effective and efficient method to improve the tissue integration and angiogenesis of subcutaneous implants.

## Introduction

Subcutaneous synthetic implants are being used in several surgical settings surgery to restore anatomy following surgical resection including breast, facial, abdominal and pelvic reconstruction. However, unacceptable levels of infection and extrusion with subcutaneous synthetic materials cause significant patient morbidity and demands on health care resources. One main reason for the poor performance of synthetic materials is the poor integration and lack of angiogenesis with the surrounding subcutaneous tissue, which leads to a lack of anchorage and consequently infection and extrusion of the implant.^1^–^3^

To improve the outcome of synthetic biomaterials, the substrate should aim to guide desired cell responses by mimicking the extracellular matrix (ECM). With the recent advancements in nanotechnology, nanocomposites offer substrates with a dimension that mimics the native ECM, improving their biocompatibility.^4^–^8^ Our group is currently developing a subcutaneous implant using a nanocomposite polyurethane.^4^,^5^ This polyurethane polymer has been extensively tested and meets international standards (ISO 10993) for biocompatibility. Due to hydrophobic nature, it has been extensively investigated for hollow organs including a vascular graft^9^ and lacrimal duct,^10^ where flow of fluids is the primary requirement rather than tissue integration.

Several methods have been investigated to modify the tissue integration of synthetic materials, including the deposition of proteins, growth factors and chemical groups.^9^–^16^ Creating functional materials is critical to modify the biological processes that occur at the material interface as they are controlled by the physiochemical properties at the material surface. Notably, manipulation of the surface chemistry and wettability of biomaterials is the simplest path for controlling surface interactions and improving biocompatibility.^17^,^18^ Biomolecules including proteins and growth factors can simply be adsorbed onto the material surface or covalently linked via chemical reactions onto the surface.^19^ Many groups have illustrated that adsorption of ECM components onto material surfaces can direct cell behavior including fibronectin, laminin, collagen and elastin.^19^ Several binding motifs from such ECMs have been identified and manufactured into short linear sequence of amino acids including RGD motifs to improve cell attachment to the biomaterial surface.^19^ However, with covalent bonding or adsorption techniques, understanding the correct presentation and gaining sufficient binding of the peptides or protein to direct cell behavior is difficult.^20^ The delivery of growth factors to direct cell behavior has also been reported by trapping the biomolecules in the scaffolds during the manufacturing process.^20^ The release kinetics of the molecule can be controlled by the porosity of the scaffolds.^20^ However, often the growth factor loses its bioactivity due to the prolonged physical entrapment.^20^ Electrostatic binding is an alternative advantageous technique as it mimics the natural interactions of the growth factors and the ECM.^20^ However, the presentation of biomolecules via this technique may not always be uniform and thus may not influence downstream pathways effectively.^20^ To overcome the challenges with using molecules to create a biometric surface, a biomaterial’s surface chemistry can be altered to direct cell behavior.^21^ Several chemical groups have been shown to influence cell behavior including carboxyl (–COOH), amine (–NH_2_), methyl (–CH3) and hydroxyl (–OH) groups.^21^ Studies have shown –NH_2_ improves adhesion, growth and matrix formation.^22^,^23^ Carboxyl groups have been shown to improve the adsorption of proteins and allow for cell adhesion.^24^ Several techniques can introduce such chemical groups onto the material surface including ion beam implantation and ion beam exchange.^25^ However, these techniques require extensive training and high costs.^26^ To overcome these challenges, an alternative method of modifying the surface chemistry of implants is using plasma surface modification (PSM).

PSM is an economical and effective method to control the physiochemical properties of biomaterials surfaces.^18^ PSM can modify the surface’s wettability by altering the surface chemistry and topography. Such physiochemical changes have been shown to enhance cell adhesion and proliferation, guide cell responses and modify the blood–biomaterial interface with the result of improving materials applicability for clinical use.^27^–^39^ An important advantage of PSM is that the treatment can be used to tune the surface chemical composition and wettability without changing the material’s bulk properties.

Radiofrequency plasma is the most widely used source of plasma, which involves passing an electric current through a gas.^18^ Various gases have been used with PSM to impart specific surface chemistries or etching effects to alter surface topography in order to tune the surface composition. Despite the extensive exploration of PSM over the years for modifying the interfacial properties of materials, there are no comprehensive studies documenting the effect of various gaseous plasmas on the characteristics of the biomaterial surface, or extensive in vitro and in vivo evaluation to confirm their efficacy. The majority of studies reported have used a variety of gases, parameters and materials, so it is difficult to compare the efficiency of various PSM methods.^18^

This study aimed to compare three commonly used gases for PSM, including argon (Ar), oxygen (O_2_) and nitrogen (N_2_), to optimize the plasma process to enhance tissue integration and angiogenesis of polyurethane scaffolds. In this study, we optimized the PSM process by treating scaffolds for various length of time, to determine the effect on polyurethane’s surface and bulk properties. The study was then expanded by in vitro and in vivo studies to find the most efficient modification process to enhance tissue integration and angiogenesis. The method developed in this study can be easily translated to other biomaterial surfaces, offering a significant advancement in developing materials for surgical applications requiring long-term subcutaneous implantation.

## Materials and methods

### Polyurethane polymer synthesis

The polymer was synthesized using a 2-part method as described previously.^11^,^12^ In brief, polycarbonate polyol (2000 molecular weight [Mw]) and trans-cyclohexanechlo-roydrinisobutyl-silses-106 quioxane (Hybrid Plastics Inc., Hattiesburg, MI, USA) were mixed followed by Polydehral Oligomeric Silsesquioxane (POSS) cages being dissolved into the polyol solution at 70°C. Then, 4, 4′-methylenebis(phenyl isocyanate) was added to form a pre-polymer. Following this, dimethylacetamide (DMAC) was then added slowly with chain extension performed by cooling the solution to 40°C. This led to the synthesis of the POSS-modified polycarbonate urea-urethane (PU) in DMAC solution. The scaffolds were manufactured using a salt-leaching method as described previously.^11^,^12^ A 3:7 wt ratio of sodium chloride (NaCl) to PU was used in all experiments. The NaCl/PU mixture was first poured onto steel molds and then placed in an oven at 65°C for 4–5 hours until all the solvent had evaporated. Casted polymer was then placed into deionized water for 7 days to remove all NaCl to produce porous scaffolds. For cell culture analysis, scaffolds were cut into circular discs of 16 mm diameter for use in 24-well plates.

### Plasma surface modification (PSM)

PSM was performed using a radio frequency plasma generator configured to 40 kHz at 100 W and a flow rate of 0.4 mbar. PSM was performed by subjecting the scaffolds to either O_2_, Ar or N_2_ gas, for 1, 2.5, 5, 7.5 or 10 minutes. Untreated (Con) scaffolds were used as the control.

### Contact angle measurements

The static water contact angle was analyzed using the sessile drop method using the DSA 100 instrument (KRUSS, Hamburg, Germany) as described previously (n=6).^11^,^12^ The static contact angle was measured on six scaffolds after varying the length of time of plasma exposure from 1 to 10 minutes.

### Mechanical testing

The mechanical properties of scaffolds after PSM were analyzed with a Instron-5565 tensile 224 tester as described previously.^11^,^12^ The Average Young’s modulus of elasticity was reported following analysis of six independent scaffolds (n=6).

### Scanning electron microscopy (SEM)

The scaffold’s surface structure and architecture, specifically the pore size and porosity (n=3), were analyzed using SEM.^11^,^12^ In brief, the scaffolds were fixed with 2.5% w/v glutaraldehyde/PBS for 48 hours. Following this, the scaffolds were critically point dried using CO_2_. The scaffolds were then gold-coated using a sc500 (EMScope; Quorum Technologies, Lewes, UK) sputter coater before imaging using a FEI Quanta 200F Scanning Electron Microscope.

### X-ray photoelectron spectroscopy (XPS) studies

The treated and untreated scaffold’s surface chemistry was evaluated using a Thermo Scientific K-Alpha spectrometer, as described previously (n=3).^11^,^12^ In brief, monochromatic Al Kα X-rays (h=1,486 eV) were focused to a 400-µm diameter spot on the scaffold surface, defining the analysis area. The analysis depth was at the photon energies 5–10 nm. Survey spectra demonstrated the elemental composition of the surface. High-resolution spectra of the principle core line of each element present were then acquired for chemical state identification. Then, high-resolution spectra were fitted with Gaussian–Lorentzian peaks using the CasaXPS software to deconvolute different chemical environments. Gaussian– Lorentzian peaks were fitted using CasaXPS software to deconvolute different chemical environments.

### Atomic force microscopy (AFM)

The roughness of the scaffolds surface was evaluated using an AFM (TAP150A) in the tapping mode (spring constant 2.919 n/m) as described previously (n=3).^11^,^12^ In brief, the root mean square roughness was calculated from the 5 µm scan of three areas using the NanoScope^®^ analysis software version 1.40 (Bruker Corporation, Billerica, MA, USA). The PeakForce quantitative nanomechanical mapping was used to obtain an FV modulus using the modified Hertzian model.

### Gel permeation chromatography (GPC)

Molecular weight averages and polymer dispersity were determined by GPC analysis. Scaffolds were prepared to 1 mg/mL concentration and passed through a 0.22 µm nylon filter before measurement. GPC measurements were performed on an Agilent 1260 infinity system equipped with 2 PLgel 5 µm mixed-D columns (300×7.5 mm), a PLgel 5 mm guard column (50×7.5 mm), a differential refractive index (DRI) and variable wavelength detector. The temperature of the columns and DRI was maintained at 50°C. The system was eluted with DMF containing 5 mM ammonium tetrafluoroborate at a flow rate of 1 mL/min and the DRI detector was calibrated with linear narrow poly(methyl methacrylate) standards with narrow molecular weight distribution (n=3).

### Differential scanning calorimetry (DSC)

The polymer’s glass transition temperature (T_g_) was measured post-PSM at 5 and 10 minutes of treatment using a Q2000 DSC (TA Instruments Ltd., Elstree, UK). Scaffolds (5–10 mg) were weighed in hermetic Tzero aluminum pans. An empty pan, matched to the weight of the scaffold pan, was used as a reference. The scaffolds were heated from 0°C to 250°C at 20°C/min. Data were analyzed with Universal Analysis 2000 with N_2_ used as the purge gas. DSC thermograms were drawn with Origin Pro 9.1 software (n=3; OrginLab, Northampton, MA, USA).

### Protein adsorption studies

#### Total protein adsorption study

Total serum protein adsorption on unmodified and modified scaffolds was determined using bicinchoninic acid assay, as described previously (n=6).^11^,^12^

#### Functional presentation of cell-binding domains of adsorbed fibronectin and vitronectin

The accessibility of cell-binding domains of bFN and bVN adsorbed on the scaffolds was examined by ELISA using monoclonal antibodies (mAbs) directed to epitopes of the RGD-containing domains as described by Barrias et al.^16^ In brief, the scaffolds were incubated in the protein solutions (bFN or bVN) of different concentrations at 37°C. After washing with PBS, all surfaces were blocked with 1% w/v BSA/PBS and then incubated with specific mouse anti-bovine mAbs at predetermined optimal concentrations (0.17 µg/mL antibFN and 1 µg/mL anti-bVN in 1% w/v BSA/PBS, Alpha Laboratories, Eastleigh, UK) for 1 hour at 37°C. After washing three times in PBS, scaffolds were incubated with horseradish peroxidase-conjugated anti-mouse IgG (H+L) secondary antibody (1:1,000 in 1% w/v BSA/PBS; Fisher Scientific, Loughborough, UK) for 1 hour at 37°C. After further washing, scaffolds were incubated in o-phenylene-diamine dihydrochloride substrate (0.4 mg/mL in 0.05 M phosphate–citrate buffer, pH 5.0, with 0.012% v/v hydrogen peroxide). The OD of the supernatant was then read using a spectrophotometer at 450 nm with reference to 620 nm.

#### Integrin-blocking experiments

The role of specific integrin involved in the fibroblast adhesion to the plasma-modified scaffolds was investigated as described by Kalaskar et al.^17^ Human dermal fibroblasts (HDFs) cells were trypsinized, counted and incubated for 30 minutes in the presence or absence of integrin-blocking antibodies α5β1 and α5β3 (Abcam, Cambridge, UK) from 1 to 10 mg/mL in cell culture medium at 37°C. The cells (treated with and without blocking antibodies) were seeded at a density of 50,000 cells/cm^3^ on the scaffolds treated with different PSMs. The number of attached cells was counted after 3 hours and cell adhesion was calculated by counting the number of attached cells in three different random fields of view in triplicate cultures.

### In vitro biocompatibility testing

#### Cell culture and cell seeding

HDFs obtained from the European Collection of Authenticated Cell Culture (ECACC) were maintained in Dulbecco’s Modified Eagle’s Medium with 10% fetal bovine serum and 1% antibiotic solutions (Sigma-Aldrich, St Louis, MO, USA) as described previously.^11^,^12^ For in vitro experiments, 16 mm scaffolds were placed into the 24-well plates, for 24 hours in complete medium before cell seeding. Each scaffold was seeded with 1×10^4^ cells/cm^2^. Every three days, the medium was changed.

#### Cellular adhesion and morphology

To study HDF architecture seeded on the scaffolds, the cytoskeleton was stained with actin-phalloidin after 24 hours.^11^,^12^ HDF viability was evaluated using Alamar Blue™ assay (Sigma-Aldrich) and the Fluorescence Hoechst DNA Quantification Kit (Sigma-Aldrich) examined DNA content on days 1, 2, 4, 7, 14 and 21 as described previously (n=6).^11^,^12^

#### Gene expression by RT-qPCR

Real-time quantitative polymerase chain reaction (RT-qPCR) was performed as described previously.^40^ First, RNA was extracted using Tri-Reagent (Life Technologies, Waltham, MA, USA) at 7 and 14 days. Second, Moloney murine leukemia virus reverse transcriptase was used to retro-transcribe the RNA (Promega, Madison, WI, USA). Following this, RT-qPCR was completed using the ABI Prism 7500 sequence detection system (Thermo Fisher Scientific, Waltham, MA, USA) with QuantiTect SYBR Green PCR kit (Qiagen, Hilden, Germany). GAPDH was used as the housekeeping gene to normalize the data using the 2^−ΔΔCt^ method. The primers used in the study are listed in Table 1.

#### Immunocytochemistry

The expression of ECM and adhesion-related proteins was using a method as previously described.^40^ At 14 days, the medium was removed and the scaffolds were washed and fixed in 4% paraformaldehyde. Following overnight incubation, the scaffolds were further washed. Following permeablization (0.5% Triton X-100), the scaffolds were blocked (0.5% BSA). Following incubation with primary antibodies overnight at 4°C, further washing was done before the scaffolds were incubated in secondary antibody for 2 hours (488 Alexa secondary antibody, 1:500). Finally, the cell nuclei were stained with Hoechst 33258 (2.5 µg/mL final concentration). Antibodies used in this study for immunocytochemistry are shown in Table 2.

#### Enzyme-linked immunosorbent assay (ELISA) of vascular endothelial growth factor (VEGF)

The VEGF secretion by the HDFs was assessed using ELISA analysis (Quantikine; R&D Systems, Inc., Minneapolis, MN, USA) according to manufacturer instructions. On days 4, 7, 10 and 14, the cell culture supernatant was taken and evaluated using ELISA as described previously.^41^

#### In vivo analysis

Following PSM treatment, two 4 mm (diameter) by 1 mm (thickness) discs were subcutaneously implanted in the dorsum of one 4-month-old BALB/c mice (Charles River Laboratories, Wilmington, MA, USA) as described previously (n=6).^11^ The disc was implanted via a small incision and closed with interrupted 5,0 monocryl. All experiments were approved by the local governmental animal care committee and performed according to animal welfare UK legislation. At 6 and 12 weeks, using CO_2_ asphyxiation, the animals were sacrificed and the scaffolds excised and fixed in 4% paraformaldehyde. The scaffolds were excised with approximately 0.5 cm surrounding circular dermis. Fixed scaffolds were then paraffin embedded and cut into 3 µm sections for histological analysis. H&E staining was conducted according to standard procedures to assess tissue integration and CD31 immunohistochemistry staining for endothelial cells detection as per a previous study.^11^ The study was approved and conducted according to the animal research UK Home Office Guidelines.

#### Statistical analysis

GraphPad (Prism) was used to conduct statistical analysis using one- or two-way ANOVA with Tukey HSD post hoc test. A *P*-value <0.05 was considered statistically significant.

## Results

### Contact angle

**Figure 1:**
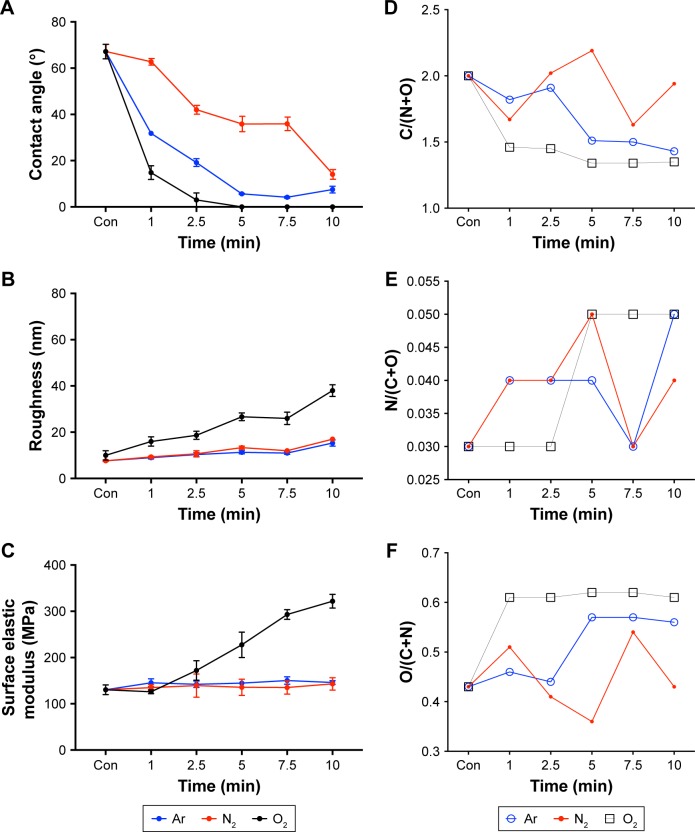
The effect of Ar, N_2_ and O_2_ plasma treatments on the (**A**) wettability (static water contact angle), (**B**) surface roughness and (**C**) surface elastic modulus of polyurethane scaffolds. **Notes:** Changes in surface properties are presented as a function of treatment time. Normalized elemental composition measured by XPS analysis for various duration plasma treatment times is presented as (**D**) ratio of carbon content, (**E**) ratio of nitrogen content and (**F**) ratio of oxygen content. **Abbreviations:** Ar, argon; Con, untreated; N_2_, nitrogen; O_2_, oxygen; XPS, X-ray photoelectron spectroscopy.

Untreated scaffolds (Con) demonstrated a static water contact angle of 67°±7°. The contact angle of the scaffolds significantly (*P*<0.05) decreased after 1 minute of Ar (32.2°±2°), N_2_ (62.7°±4°) and O_2_ (14.8°±7°) treatment compared with unmodified scaffolds (Con; Figure 1A).

### Roughness using AFM

PSM affected the surface roughness of the scaffolds following Ar, O_2_ and N_2_ treatment. Unmodified scaffolds (Con) had a roughness of 8±1 nm (Figure 1B). Changes in surface roughness following Ar and N_2_ were only significant compared with control after 5 minutes of treatment time (N_2_, 1 minute, 9±1 nm; N_2_, 5 minutes, 13±2 nm; Ar, 1 minute, 9±1 nm; Ar, 5 minutes, 11±2 nm). However, O_2_ plasma led to linear increase in surface roughness with treatment time (O_2_, 1 minute, 16±3 nm; O_2_, 10 minutes, 38±4 nm), suggesting it was the most aggressive plasma treatment, and such changes were confirmed using SEM (Figure S1).

### Surface elastic modulus using AFM

Ar and N_2_ plasma treatment showed no change in the surface elastic modulus as the treatment time increased compared with control (Con; Figure 1C). However, with O_2_ treatment, the surface elastic modulus increased linearly as the treatment time increased (*P*<0.05).

### XPS measurements

For XPS analysis, the relative molar concentrations of C, N and O from all scaffolds were normalized and presented as shown in Figure 1D–F. The C/(N+O) ratio showed a stepwise decrease after treatment with Ar and O_2_. It is suggested that this trend may be related to the removal of residual carbon contamination from the surface (Figure 1D). Conversely, the C/(N+O) ratio did not follow any definite pattern after the N_2_ treatment. The observed trend suggests there is a cyclic removal and deposition of carbon from the surface of the material under such conditions.

On the other hand, the N/(C+O) ratio only showed small changes after treatment with all three gases (Figure 1E), suggesting no significant changes to surface N concentration for all the scaffolds. For the O/(C+N) ratio, a significant increase was observed during the O_2_ treatment after one minute (Figure 1F). Further scaffold treatment did not bring an increase of the O/(C+N) ratio. For the Ar treatment, the O/(C+N) ratio increased gradually until five minutes, which remained constant even after longer treatment times. N_2_ treatment did not follow any particular trend. The calculated ratio suggests a similar pattern as that observed for the C/(N+O) treated with N_2_ plasma. Overall, the C ratio decreases with Ar and O_2_ treatment, and in response, the O ratio increases with Ar and O_2_ treatment. N_2_ treatment, on other hand, does not seem to have a significant effect over the polymer chemistry during the treatment process.

### Bulk properties including mechanical testing, DSC analysis and molecular weight analysis

The changes in bulk properties of the scaffolds after PSM were examined by the GPC, DSC and mechanical properties. After 5 minutes of treatment, irrespective of the type of gas, there were no significant changes found in average molecular number (M_n_) or average molecular weight (Mw) using GPC (Figure S2). After modification with plasma treatment, there was no difference in the Young’s modulus of the scaffolds with either Ar, N_2_ or O_2_ compared with untreated scaffolds (Con; Figure S3). There were no significant changes in the T_g_ after different PSM techniques using DSC analysis (Figure S4).

### HDF adhesion

**Figure 2:**
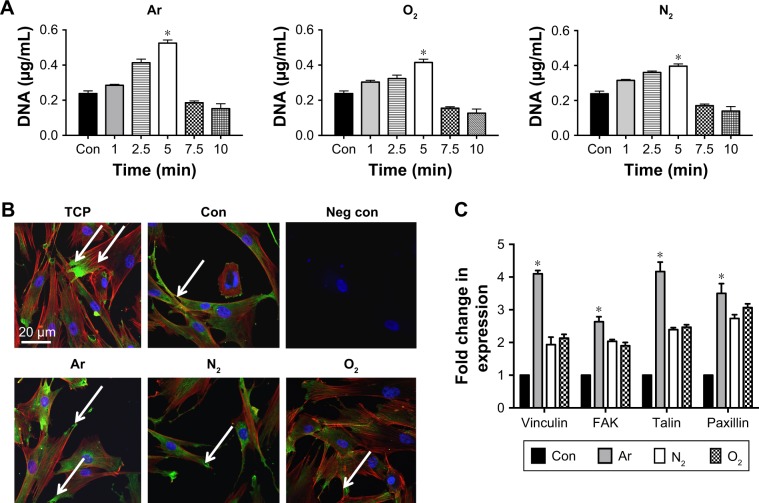
Attachment of the HDF on the scaffolds treated with plasma surface modification after 24 hours. **Notes:** (**A**) DNA assay for various treatment times using Ar, N_2_ and O_2_ plasma treatment shows the highest level of cell attachment post 5 minutes of PSM treatment. (**B**) The effect of plasma modification on actin cytoskeleton organization and vinculin expression shows cell spreading on all scaffolds, with evidence of FAKs being formed after 24 hours (white arrows) (green: vinculin staining; red: actin filaments and blue: nucleus staining using DAPI). (**C**) Quantification of gene expression of adhesion-related genes, vinculin, FAK, talin and paxillin shows significant upregulation (*P<0.05), on Ar-modified scaffolds compared with other plasma gases and untreated scaffolds, after 5 minutes of treatment. Fold change represents the differences compared with housekeeping gene GAPDH of cells grown on unmodified scaffolds (Con). **Abbreviations:** Ar, argon; Con, untreated; FAK, focal adhesion kinase; HDF, human dermal fibroblast; N_2_, nitrogen; O_2_, oxygen; TCP, tissue culture plate; Neg con, negative control where primary antibody was omitted.

For all the gaseous plasma, 5 minutes provided the highest cellular attachment (Figure 2A). However, among all the test gases for 5 minutes, the highest cell adhesion at 24 hours was observed on Ar-modified scaffolds compared with O_2_- and N_2_-modified scaffolds (*P*<0.05).

When cell morphology was examined by staining cytoskeleton of the cells, HDF cells demonstrated spreading on all plasma-modified scaffolds and unmodified scaffolds (Figure 2B). In addition to this, HDF vinculin expression was confirmed on all scaffolds by immunocytochemistry after 24 hours. The expression of vinculin, focal adhesion kinase (FAK), talin and paxillin was higher on the PSM scaffolds compared with unmodified scaffolds (*P*<0.05). Among the gases tested, the expression of adhesion-related genes was significantly higher (*P*<0.05) on Ar compared with unmodified, N_2_ and O_2_ scaffolds (Figure 2C).

### HDF viability and growth

**Figure 3:**
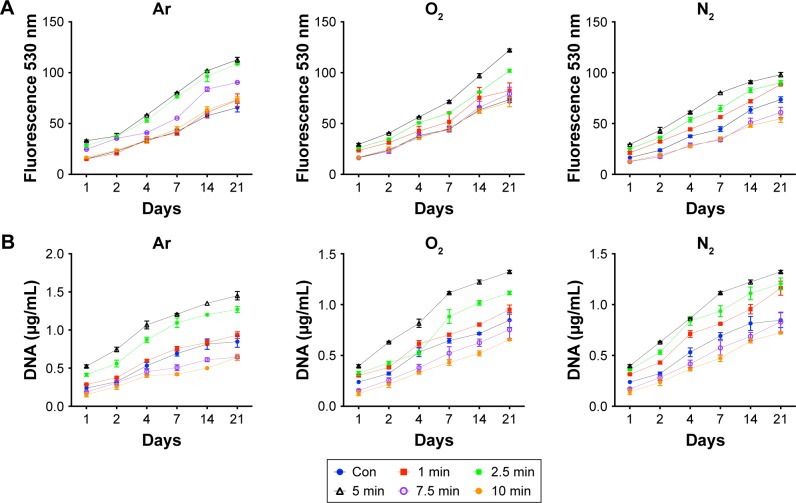
Human dermal fibroblast viability (**A**) and DNA (**B**) over 21 days on the scaffolds treated with plasma surface modification. **Note:** Viability was measured using Alamar Blue and DNA assay, respectively, for Ar, N_2_ and O_2_ plasma for various treatment times (n=6) compared with untreated scaffolds (Con). **Abbreviations:** Ar, argon; Con, untreated; N_2_, nitrogen; O_2_, oxygen.

Short-term cellular viability, determined using Alamar Blue assay and DNA content analysis, was significantly higher on plasma-modified scaffolds compared with unmodified control scaffolds with all treatment gases (*P*<0.05; Figure 3). For all treatment gases, the greatest level of cell growth was observed after 5 minutes of treatment compared with other treatment times.

### Total protein adsorption

**Figure 4:**
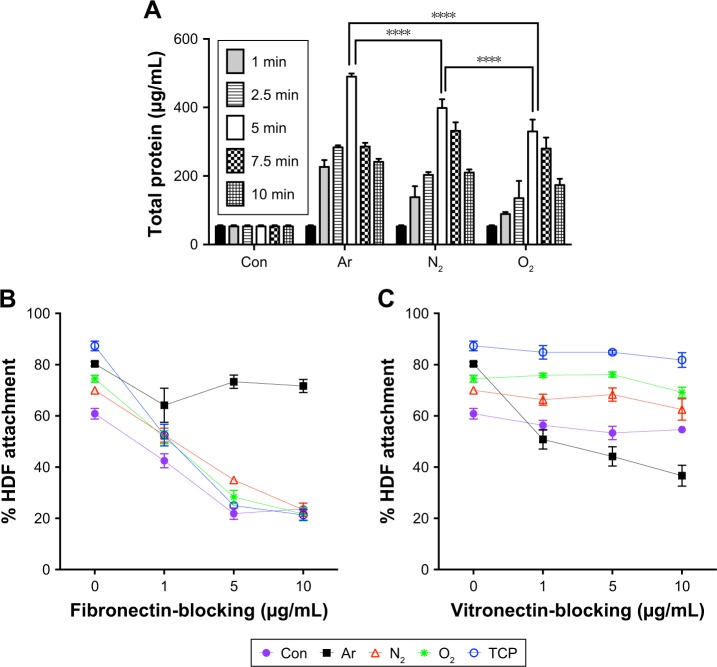
Protein adsorption studies. (**A**) Total protein adsorption on the scaffolds after plasma surface modification. Total serum protein adsorption was evaluated using the BCA assay. Total serum protein adsorption on all scaffolds was significantly higher after 5 minutes of PSM for all the test gases (****P<0.0001). Ar shows the highest protein adsorption among all the scaffolds (n=6). (**B**) Fibronectin and (**C**) vitronectin integrin-blocking experiments to assess specificity of adsorbed proteins on the scaffolds treated with plasma surface modification. **Notes:** Blocking of the fibronectin receptor α_5_β_1_ using fibronectin-blocking antibody (Anti-FN) led to dose-dependent decreased cell adhesion on the N_2_- and O_2_-treated scaffolds but not on Ar-treated scaffolds, confirming the role of fibronectin-mediated attachment on N_2_- and O_2_-treated scaffolds. Blocking of the vitronectin receptor α_V_β_3_ using vitronectin-blocking antibody (Anti-VN) led to dose-dependent decreased cell adhesion on the Ar-treated scaffold but not on N_2_- and O_2_-treated scaffolds, confirming the dominant role of vitronectin-mediated attachment on Ar scaffolds (n=6). **Abbreviations:** Ar, argon; BCA, bicinchoninic acid; Con, untreated; N_2_, nitrogen; O_2_, oxygen; PSM, plasma surface modification; TCP, tissue culture plate.

The type, amount and conformation of the essential proteins at surface interface determine the cell adhesion, their attachment and long-term growth. In order to understand the increase in growth of HDF cells on the PSM scaffolds, total protein adsorption was quantified on all scaffolds (Figure 4A). PSM scaffolds showed significantly higher amount of total protein adsorption compared with unmodified scaffolds. Among various treatment times used, 5 minutes of treatment with all test gases plasma led to the highest protein adsorption from serum containing media compared with control. Furthermore, when various test gases are compared, Ar (5 minutes) showed the highest protein adsorption compared with N_2_ and O_2_. To analyze the role of specific proteins in cell adhesion, the most common ECM proteins, FN and VN, adsorption was quantified (Figure S5). FN adsorption was found to be the highest on N_2_- and O_2_-treated scaffolds along with tissue culture plate (TCP; Figure S5), which reached saturation after 5 µg/mL, whereas, VN adsorption was found to be the highest on Ar along with TCP (Figure S5). Blocking integrin receptors for VN and FN demonstrated that the fibroblasts adhered to the modified surfaces using different receptors (Figure 4B and C). Fibroblasts adhered to N_2_- and O_2_-modified surfaces using fibronectin receptor α_5_β_1_ but using vitronectin receptor α_V_β_3_ on Ar-modified surfaces. Since 5 minutes of treatment showed the highest protein adsorption, followed by cell viability and growth with all the gases, this treatment time was further used to understand the mechanism of cellular interaction in terms of role of specific protein adsorption, interaction of cell surface receptors and their effect on tissue integration and angiogenesis.

### ECM formation in vitro

**Figure 5:**
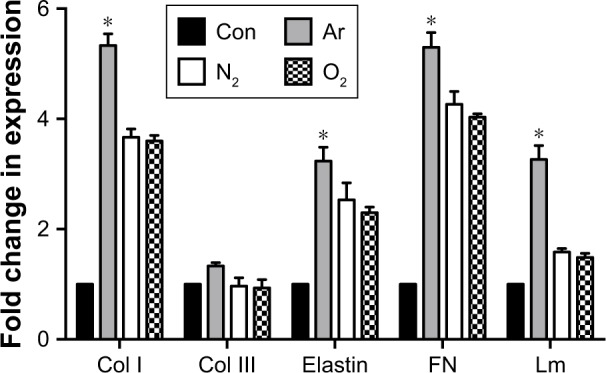
Gene expression of ECM markers by the human dermal fibroblasts on plasma-modified scaffolds using RT-qPCR. **Notes:** Gene expression confirmed the upregulation of ECM markers significantly on the Ar-modified scaffolds after 14 days compared with other scaffolds (*P<0.05). All scaffolds were treated with 5 minutes of respective plasma surface modification. Fold change represents the differences compared with housekeeping gene GAPDH of cells grown on unmodified scaffolds (Con). *P<0.05. **Abbreviations:** Ar, argon; Col I, collagen type I; Col III, collagen type III; Con, untreated; ECM, extracellular matrix; FN, fibronectin; Lm, laminin; N_2_, nitrogen; O_2_, oxygen; RT-qPCR, real-time quantitative polymerase chain reaction.

The mRNA expression and release of ECM factors of collagen type I, elastin, laminin and fibronectin were greater on Ar-modified scaffolds than on N_2_- and O_2_-modified scaffolds (*P*<0.05; Figure 5). There was no difference in collagen type III expression by the HDFs between the scaffolds (Figure 5). The protein expression of collagen, elastin and fibronectin by the HDFs was confirmed on the scaffolds using immunocytochemistry after PSM with all gases, as well as on unmodified scaffolds (Figure S6).

### Assessment of tissue integration and angiogenesis in vivo

**Figure 6:**
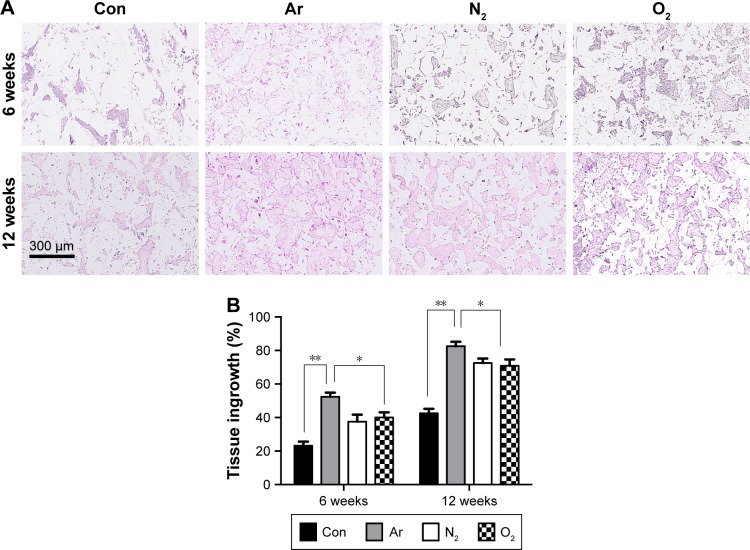
Tissue integration of the plasma-modified scaffolds in a subcutaneous implantation mouse model for 6 and 12 weeks. **Notes:** (**A**) Tissue ingrowth was found to be increased after 6 and 12 weeks on Ar-modified scaffolds compared with N_2_- and O_2_-modified scaffolds and unmodified (Con) scaffolds using H&E staining. Tissue integration for O_2_- and N_2_-modified scaffolds demonstrated similar levels. Scale bar: 300 µm. (**B**) Quantification revealed Ar with the highest tissue ingrowth compared with other scaffolds. All scaffolds were treated with 5 minutes of respective plasma surface modification. *P<0.05, **P<0.01. **Abbreviations:** Ar, argon; Con, untreated; N_2_, nitrogen; O_2_, oxygen.

**Figure 7:**
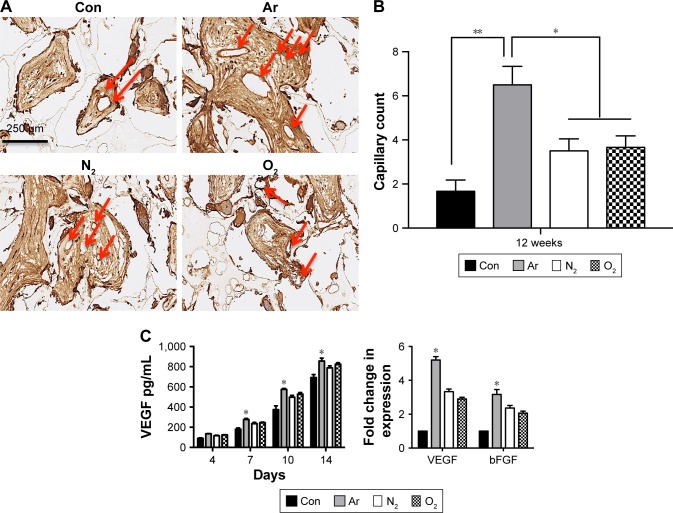
Angiogenesis of the plasma-modified scaffolds after five minutes of treatment. **Notes:** (**A**) Angiogenesis assessment after 12 weeks in a mouse model. Vessel ingrowth was identified using CD31 marker, where the number of capillaries was identified (red arrows). Scale bar: 250 µm. (**B**) Quantification showed a significantly higher capillary count on Ar-modified scaffolds compared with N_2_ and O_2_-modified scaffolds. (**C**) Analysis of the secretion of VEGF and bFGF by human dermal fibroblasts. Left: VEGF secretion measured by ELISA demonstrates increased protein secretion on the Ar-modified scaffolds at 7, 10 and 14 days by the HDFs compared with N_2_ and O_2_-modified scaffolds and unmodified scaffolds (Con). Right: The mRNA expression of VEGF and bFGF at 14 days was increased on the Ar-modified scaffolds compared with N_2_- and O_2_-modified scaffolds. Fold change represents the differences in the expression of the housekeeping gene GAPDH between cells grown on unmodified scaffolds (Con) and modified scaffolds. *P<0.05, **P<0.01. **Abbreviations:** Ar, argon; bFGF, basic fibroblast growth factor; Con, untreated; N_2_, nitrogen; O_2_, oxygen; VEGF, vascular endothelial growth factor.

To assess the ability of the scaffolds to support tissue integration in vivo, the scaffolds were subcutaneously implanted for 6 and 12 weeks in a mice model and levels of tissue integration and angiogenesis were compared. Figure 6A shows the histology and immunohistochemistry of the explanted scaffolds treated with the three different gases and unmodified scaffolds. Quantification of H&E staining confirms significantly higher level of tissue ingrowth in the Ar-modified scaffolds compared with O_2_-, N_2_-treated and unmodified scaffolds at 6 and 12 weeks (*P*<0.05; Figure 6B). The degree of capsule formation was also measured after 12 weeks and found to be significantly reduced after PSM compared with unmodified scaffolds (Figure S7). Vascularization in vivo was assessed using CD31 staining to identify capillaries growing into the scaffolds after different PSM (Figure 7A). Analysis indicated that Ar-modified scaffolds had greater vessel formation than other plasma-modified scaffolds and unmodified scaffolds (*P*<0.05; Figure 7B). Furthermore, in vitro quantification of the secretion and expression of angiogenic growth factors, vascular endothelial growth factor (VEGF) and basic fibroblast growth factor, was also higher on Ar-modified scaffolds compared with N_2_-and O_2_-modified scaffolds (Figure 7C).

## Discussion

Surgical implants fail due to poor integration and lack of vascularization with the surrounding tissue.^2^ In this study, we used a cytocompatible material polyurethane to overcome that challenge by modifying it with PSM. Even though the usefulness of PSM has been widely documented, there is a lack of information on the efficiency of various gases used in PSM and optimal process parameters, which can be used to enhance tissue integration and angiogenesis of synthetic implants.

From the surface and bulk analyses, it is clear that polymer bulk properties remain unaffected by plasma treatment for various length of time and only the surface interfacial properties were altered. This is in agreement with previous reports, which suggest, that PSM has no effect on the bulk properties of materials.^11^,^42^,^43^ Detailed surface analysis revealed that all treatment gases significantly decreased the surface wettability, and led to an increase in topography and surface elastic modulus of the PSM scaffolds compared with unmodified scaffolds (Figure 1; *P*<0.05). The O_2_-treated scaffolds demonstrated an increasing roughness and surface elastic modulus as treatment time increased. However, on the other hand, N_2_ and Ar treatment showed very small changes to the roughness and the surface elastic modulus over 10 minutes. The surface wettability of the scaffolds was reversed by PSM after 1 minute of Ar, N_2_ and O_2_ treatments. The O_2_ surface modification also showed a greater effect on decreasing the contact angle of the scaffolds after similar exposure rates of Ar and N_2_ PSM.

The decrease in the contact angle and the increase in the surface roughness following O_2_ plasma are consistent with previous reports.^27^ Pappa et al found that O_2_ plasma significantly decreased the contact angle and increased the surface roughness of polycaprolactone polymer surfaces due to the introduction of polar groups on the surface of the polymer.^27^ Shah et al also demonstrated similar decreases in contact angle findings following the Ar and N_2_ treatment of polylactic acid surfaces.^30^ N_2_ has been shown to cause hydrophilic surfaces due to the deposition of –NH_2_ chemical functional groups.^36^ Ar has been shown to create hydrophilic surfaces due to deposition of polar functional groups (–OH) groups.^33^ In this study, we found a small increase in roughness after 5 minutes of Ar and N_2_ plasma treatment. Previous studies have shown both an increase and decrease in surface roughness following Ar and N_2_ plasma treatment.^30^,^32^,^37^,^39^ The differences in the literature may be due to varying intensities of the plasma gases generated due to contrasting methodology within studies.^30^

The XPS analysis confirmed that Ar and O_2_ plasma treatment acted as an effective carbon remover. A decrease in the carbon ratio and increase in the N_2_ ratio seemed proportional with these two treatment gases. As carbon was removed, underlying N_2_ from scaffolds surface was possibly exposed. It should also be noted that part of detected carbon could be associated to adventitious contamination, which is commonly observed in XPS analysis. Ar and O_2_ plasma treatments also showed an increase in O_2_ deposition. On the other hand, N_2_ treatment showed a pattern that could be demonstrating cyclical N_2_ deposition, carbon removal and O_2_ deposition from the surface, suggesting that there was very little effect on surface chemistry. Furthermore, this also shows that N_2_ plasma caused the smallest change to the scaffold’s surface chemistry. Taking all surface analysis results into consideration, it is safe to suggest that among all three gases tested, O_2_ treatment brought the most significant changes, Ar showed moderate changes and N_2_ treatment had the least effect on the surface properties. These changes may be accounted for by the difference in the reactivity of the plasma gas.^30^ The treatment of reactive gases like O_2_ brings about more surface changes than less reactive gases such as Ar and N_2_.^30^

The changes in bulk properties post-PSM were investigated in terms of mechanical properties, Mw and Mn, and T_g_ after increasing exposure of Ar, O_2_ and N_2_ treatment. The tensile mechanical properties and T_g_ of the polymer did not change after 10 minutes of Ar, O_2_ or N_2_ treatments. However, GPC showed a decrease in the Mw and Mn after 10 minutes of O_2_ modification, suggesting a time less than 10 minutes should be used to prevent the degradation of nanocomposite materials when using this treatment gas. The surface elastic modulus increased after increasing exposure of O_2_ treatment, which could be due to oxidative degradation and repolymerization of polymer forming harder interfaces.^44^ Tiganis et al demonstrated that an increase in the Young’s modulus at the specimen surface of acrylonitrile butadiene styrene using micro-indentation measurements indicated degradation of the polymer causing the polymer to become brittle and fail.^44^ However, further analysis and reports into polymer degradation are needed to account for this observation.

To find optimum cell culture conditions, HDF cells were cultured on PSM-treated polymers for various treatment times. It was interesting to note that, after 5 minutes of exposure, all PSM treatments enhanced the adhesion (Figure 2A) and growth (Figure 3A and B) of HDFs on the scaffolds. Cells do not directly interact with synthetic materials but attach to the absorbed layer of proteins on the material surface. Protein adsorption was greater on the PSM surface, correlating with enhanced cell adhesion on modified compared with unmodified scaffolds (Figure 4A). It is hypothesized that although there was low protein adsorption on unmodified scaffolds, the conformation was correct allowing for spreading of the fibroblasts similar to plasma-modified scaffolds, but the lower protein adsorption resulted into low levels of cell attachment. However, when the surfaces were modified with Ar, O_2_ or N_2_, there was more protein available causing higher cell attachment and cell spreading (Figure 4A). Among three gases for treatment, Ar showed the highest protein adsorption and thus cell attachment. To understand the reason behind this, further analysis of the cell–protein interaction was undertaken.

Cells recognize adsorbed proteins via integrins, which are linked between the ECM and the actin cytoskeleton.^45^,^46^ Integrins form focal adhesion complexes when cells are adhered. Such complexes contain structural proteins including vinculin, α-actin and talin, and signaling molecules such as paxillin and FAK.^46^,^47^ We observed an increase in the expression of vinculin, paxillin, talin and FAK by the HDFs on Ar-modified scaffolds. This may have contributed to the increase in cell adhesion and long-term growth observed on Ar-modified scaffolds compared with other PSM scaffolds.

Interestingly, this study also found that adhesion of the HDFs to the plasma-modified scaffolds occurred through different integrins. The integrin-blocking experiment showed a selective decrease in cell attachment on O_2_ and N_2_ when anti-α_5_β_1_-integrin antibody was used. This observation confirms the role of fibronectin on these surfaces, which is known to interact with cells via its classical α_5_β_1_ integrin receptor.^46^ But the decrease in cell attachment was only observed on Ar-treated scaffolds, when anti-α_V_β_3_-integrin antibody was used, suggesting a predominate role of vitronectin in cellular attachment on these scaffolds.^47^ However, as the percentage decrease in cell attachments was around 40% with anti-α_V_β_3_-integrin antibody on Ar-treated scaffolds, role of other cell surface receptors cannot be overlooked.

The data presented in this study show that the PSM scaffolds provide an optimum interface in terms of chemistry and topography after 5 minutes of treatment. The changes in the interfacial surface of polymer facilitated an optimum concentration and conformation of selective adhesion proteins, which resulted in enhanced cell attachment, growth and cellular function. It is possible that these interfacial changes induced by Ar allowed the highest number of proteins to be adsorbed in their optimal conformation due to moderate changes in surface hydrophilicity, roughness and surface chemistry. These surface changes resulted in higher cell attachment, growth and cellular function compared with O_2_ and N_2_ treatments. Figure S8 schematically explains this concept in more detail.

The observed short-term cell behavior was maintained in the long-term culture. The long-term cell survival and growth of HDF cells also led to the upregulation of several ECM-related genes including collagen type I, fibronectin, elastin and laminin (Figure 5). HDFs on Ar-treated scaffolds continued to express the highest level of ECM-related genes and also deposit corresponding ECM proteins (Figure S6). This confirmed that PSM enhances cellular function not only in the short term but also in long-term in vitro culture. Previous studies using PSM also showed an increase in the ECM production for other anchorage-dependent cells such as osteoblasts and chondrocytes.^48^,^49^

To ascertain the observed cellular behavior is not an effect of in vitro culture, further in vivo studies were conducted using mice model. Histological analysis of explanted scaffolds after 6 and 12 weeks showed a significantly higher level of tissue integration on PSM scaffolds compared with untreated scaffolds. However, it was highest on Ar-modified scaffolds compared with O_2_- and N_2_-treated scaffolds. Fibrous capsule formation around synthetic implants during in vivo studies is a common phenomenon. Examining the extent of fibrous capsule thickness can serve as good indicator of tissue material reaction. This study showed that the thickness of fibrous capsule formation on PSM scaffolds is significantly reduced compared with untreated scaffolds (Figure S7), suggesting that PSM not only facilitates tissue integration but also minimizes the inflammatory reactions.

The development of new blood vessels after clinical implantation of a material for restoration of facial defects is a limiting factor in all three-dimensional bioengineered constructs. Many growth factors are responsible for angiogenesis. Ring et al illustrated that dermal substitutes and allogenic bone implants enhanced their vascularization after Ar/hydrogen and Ar/O_2_ plasma treatment, respectively, using a dorsal skinfold chamber assay.^50^,^51^ In the current study, all plasma treatments led to increase in angiogenesis compared with untreated scaffolds. Ar treatment revealed the highest vessel formation as shown by CD31 staining (Figure 7A and B). During in vitro culture, HDFs secreted the highest amounts of VEGF (Figure 7C), which might have supported a greater influx and proliferation of endothelial cells and allowing for greater vessel formation in vivo.

In summary, to the best of our knowledge, this study for the first time reports a detailed investigation into the effect of various gases used for PSM on polymer surfaces and their effect on the short- and long-term tissue integration and angiogenesis. PSM significantly affected the surface chemistry of the polyurethane polymer, without affecting its bulk properties. Optimal PSM time for the polyurethane polymer was found to be 5 minutes based on both in vitro and in vivo studies for all the gases tested (Ar, O_2_ and N_2_). However, Ar modification was optimal for the polyurethane scaffolds in this study for the enhancement of cellular integration and angiogenesis. This is due to the unique combination of surface chemistry and topographical modification this treatment gas imparts.

## Conclusion

Ar PSM is a simple, fast and very effective method of surface modification that can improve tissue integration and angiogenesis of polyurethane implants. Future work will investigate the effect of Ar surface modification on further clinically approved biomedical materials to assess its effectiveness at the clinical bedside.

## Supplementary materials

Figure S1 SEM images of the surface of the scaffolds modified with Ar, N_2_ and O_2_ using plasma surface modification for various length of time.
**Note:** Scale bar: 2 µm.
**Abbreviations:** Ar, argon; Con, untreated; N_2_, nitrogen; O_2_, oxygen; SEM, scanning electron microscopy.

Figure S2 GPC after 5 and 10 minutes of plasma surface modification using Ar, N_2_ and O_2_ treatment.
**Notes:** (**A**) Effect of plasma modification on Mw. (**B**) Effect of plasma modification on Mn. (**C**) Effect of plasma modification on PDI. No significant changes were found after 5 or 10 minutes of Ar or N_2_ treatment. Ten minutes of O_2_ treatment caused a significant decrease in the Mw and Mn compared with unmodified scaffolds (*P*<0.05). **P<0.01.
**Abbreviations:** Ar, argon; Con, untreated; GPC, gel permeation chromatography; Mn, molecular number; Mw, molecular weight; N_2_, nitrogen; O_2_, oxygen; PDI, polydispersity index.

Figure S3 Mechanical properties of the scaffolds after plasma surface modification.
**Note:** Plasma surface modification showed no change in the tensile Young’s elastic modulus for all exposure times (up to 10 minutes).
**Abbreviations:** Ar, argon; Con, untreated; N_2_, nitrogen; O_2_, oxygen.

Figure S4 DSC after 5 and 10 minutes of plasma surface modification using Ar, N_2_ and O_2_ treatment.
**Note:** This study showed no changes in the T_g_ after 5 and 10 minutes of plasma surface modification using Ar, N_2_ or O_2_ compared to untreated scaffolds (Con).
**Abbreviations:** Ar, argon; DSC, differential scanning calorimetry; N_2_, nitrogen; O_2_, oxygen; T_g_, glass transition temperature.

Figure S5 Functional presentation of cell-binding domains of adsorbed fibronectin and vitronectin after plasma surface modification. Total fibronectin (**A**) and (**B**) vitronectin protein adsorption onto the scaffolds after 1 hour with 5 minutes of plasma surface modification exposure was analyzed using mAB.
**Notes:** After 5 minutes of N_2_ or O_2_ modification, greater amount of fibronectin was absorbed onto the scaffolds compared with Ar-modified and unmodified scaffolds (Con). Vitronectin adsorption was the greatest after 5 minutes of Ar modification compared with all other scaffolds.
**Abbreviations:** Ar, argon; Con, untreated; mAB, monoclonal antibodies; N_2_, nitrogen; O_2_, oxygen; TCP, tissue culture plate.

Figure S6 ECM formation by the human dermal fibroblasts after plasma surface modification. Immunocytochemistry confirmed the expression of the ECM markers after 14 days on the plasma-modified and untreated scaffolds (green: collagen type I, elastin, fibronectin; blue: DAPI).
**Note:** Scale bar: 20 µm.
**Abbreviations:** Ar, argon; Con, untreated; ECM, extracellular matrix; N_2_, nitrogen; Neg con, negative control where primary antibody was omitted; O_2_, oxygen; TCP, tissue culture plate.

Figure S7 Fibrous capsule formation of the scaffolds treated with plasma surface modification after 12 weeks of subcutaneous implantation.
**Notes:** (**A**) Histological sections representing thickness of fibrous capsule at the interface of implant and subcutaneous tissue. Scale bar: 200 µm. (**B**) Quantification of fibrous capsule thickness shows significantly higher fibrous capsule thickness on the unmodified scaffolds compared with plasma-treated scaffold (*P<0.05).
**Abbreviations:** Ar, argon; Con, untreated; N_2_, nitrogen; O_2_, oxygen.

Figure S8 Schematic representation of the mechanism by which cells interact with unmodified and plasma surface-modified scaffolds using Ar, N_2_ and O_2_ plasma treatments.
**Notes:** Control scaffolds show lower protein adsorption and cell attachment, Ar-modified scaffolds show moderate roughness, with higher VN adsorption from serum protein, N_2_ plasma-modified scaffolds show higher FN adsorption compared with O_2_ plasma-modified scaffolds, which exhibit the highest surface roughness. Image not to the scale.
**Abbreviations:** Ar, argon; FN, fibronectin; N_2_, nitrogen; O_2_, oxygen; VN, vitronectin.

## Figures and Tables

**Table 1:** The RT-qPCR primer sequences used in this study

Gene of interest	Primer sequence	Annealing temperature°	Cycles (RT-PCR)
Collagen type I	F ATGCCTGGTGAACGTGGT	56	26
	R AGGAGAGCCATCAGCACCT		
Collagen type III	F TGGATCAGGCCAGTGGAAAT	59	35
	R AGTGTGTTTCGTGCAACCAT		
Fibronectin	F GAGTAAACCTGAAGCTGAAGAGACT	56	35
	R GCATGATCAAAACACTTCTCAGCTA		
Elastin	F CAGGTGCGGTGGTTCCTC	59	35
	R CACCTACACCTGGAGCCTTG		
Laminin	F TCTGACCTCAACCGCCTAGA	59	35
	R CGGATGGGCTCCAGTAAACA	59	
Focal adhesion kinase (FAK)	F CAGGGTCCGATTGGAAACCA	59	35
	R AAGCTTGACACCCTCGTTGT		
Vinculin	F CCCCTGACATGGAAGACGATT	59	35
	R TGTCATTGCCCTTACTAGACCAC		
Paxillin	F CATGGACGACCTCGACGC	59	35
	R CAAGAACACAGGCCGTTTGG		
Talin	F GCAGCTTACCACCCAGAAGT	59	35
	R TCTGCAGGGTCAGCAGTACAT		
Vascular endothelial growth factor (VEGF)	F TCATCACGAAGTGGTGAAGTTCAT	59	35
	R TACTCCTGGAAGATGTCCACCA		
Basic fibroblast growth factor (bFGF)	F TCCACCTATAATTGGTCAAAGTGGT	59	35
	R CATCAGTTACCAGCTCCCCC		
Glyceraldehyde 3-phosphate dehydrogenase (GAPDH)	F TGATGACATCAAGAAGGTGGTGAAG	56	28
	R TCCTTGGAGGCCATGTGGGCCAT		

**Abbreviation:** RT-PCR, real-time polymerase chain reaction.

**Table 2:** Primary antibody details used in this study

Antibody	Host	Manufacturer	Dilution	Incubation
Vinculin	Mouse	Abcam (ab18058)	1:200	Overnight
				4°C
Elastin	Rabbit	Abcam (ab21607)	1:200	Overnight
				4°C
Collagen type I	Rabbit	Abcam (ab34710)	1:200	Overnight
				4°C
Fibronectin	Rabbit	Abcam (ab2413)	1:200	Overnight
				4°C
