# Supplementary figures and images for: Argon plasma improves the tissue integration and angiogenesis of subcutaneous implants by modifying surface chemistry and topography
Source: Int J Nanomedicine. 2018 Oct 8;13:6123–41. doi: 10.2147/IJN.S167637 (PMC6181122; doi:10.2147/IJN.S167637)

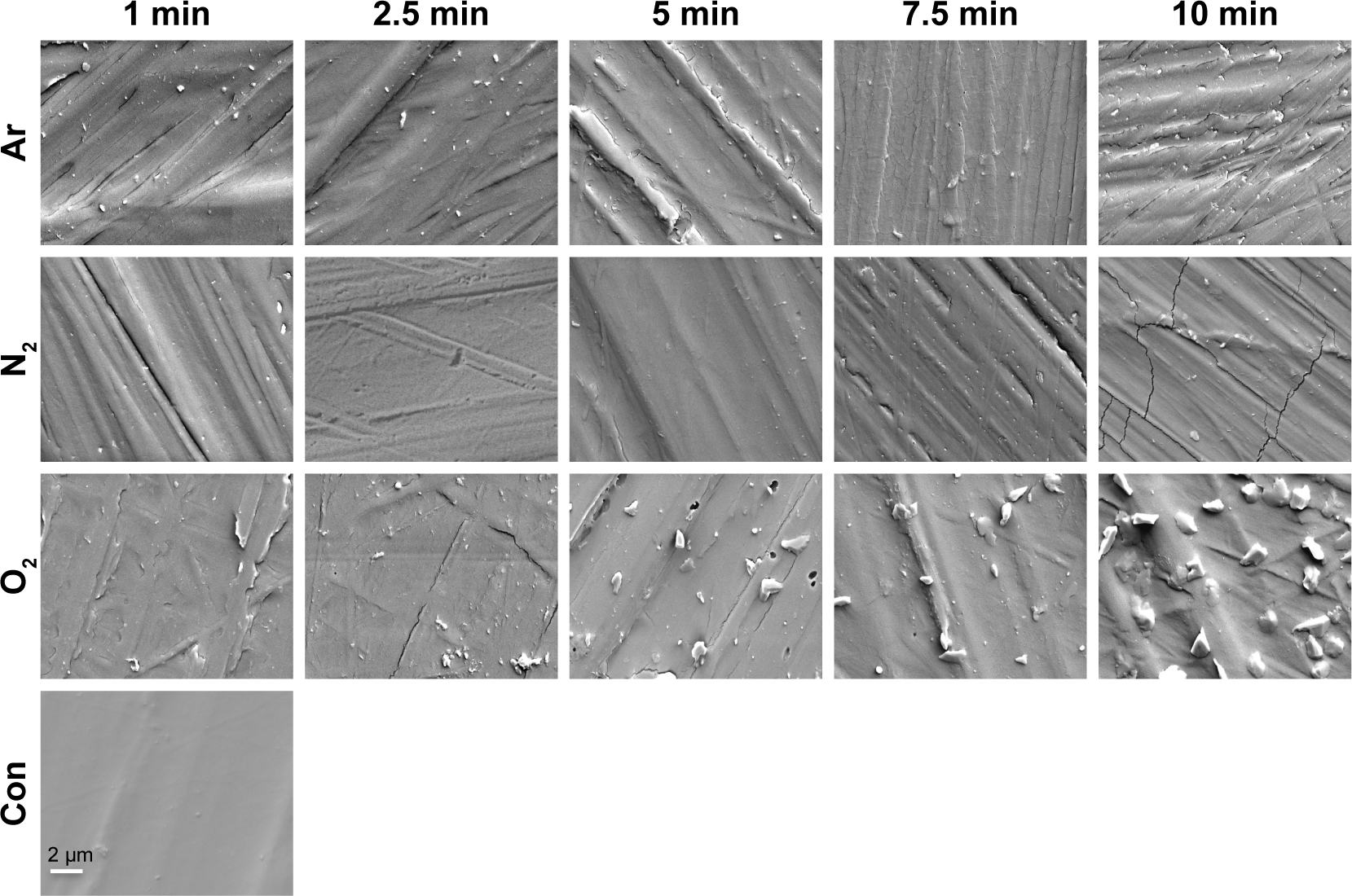

Supplement: Figure S1 — SEM images of the surface of the scaffolds modified with Ar, N2 and O2 using plasma surface modification for various length of time. Note: Scale bar: 2 µm. Abbreviations: Ar, argon; Con, untreated; N2, nitrogen; O2, oxygen; SEM, scanning electron microscopy. [file ijn-13-6123s1.tif]

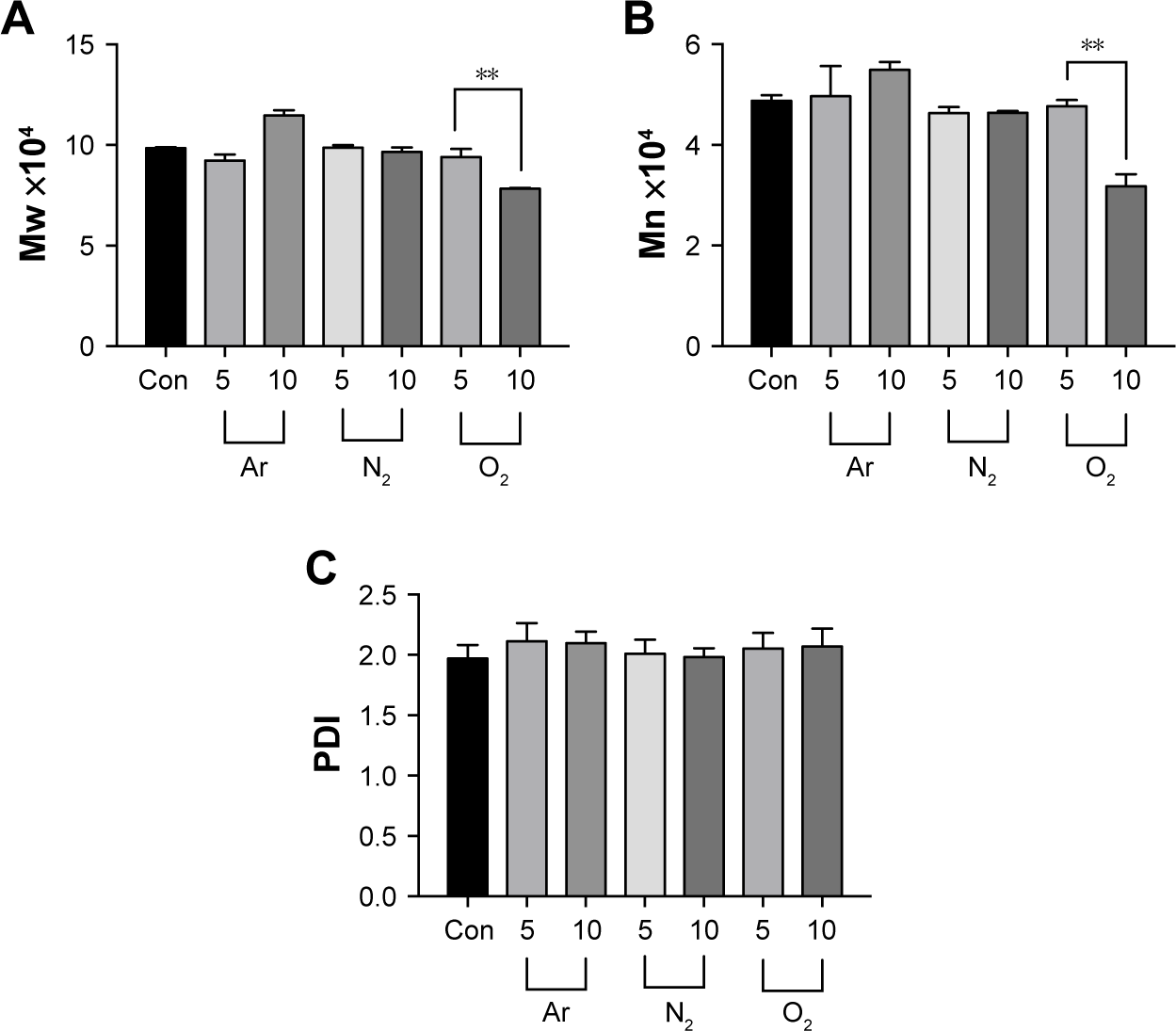

Supplement: Figure S2 — GPC after 5 and 10 minutes of plasma surface modification using Ar, N2 and O2 treatment. Notes: (A) Effect of plasma modification on Mw. (B) Effect of plasma modification on Mn. (C) Effect of plasma modification on PDI. No significant changes were found after 5 or 10 minutes of Ar or N2 treatment. Ten minutes of O2 treatment caused a significant decrease in the Mw and Mn compared with unmodified scaffolds (P<0.05). **P<0.01. Abbreviations: Ar, argon; Con, untreated; GPC, gel permeation chromatography; Mn, molecular number; Mw, molecular weight; N2, nitrogen; O2, oxygen; PDI, polydispersity index. [file ijn-13-6123s2.tif]

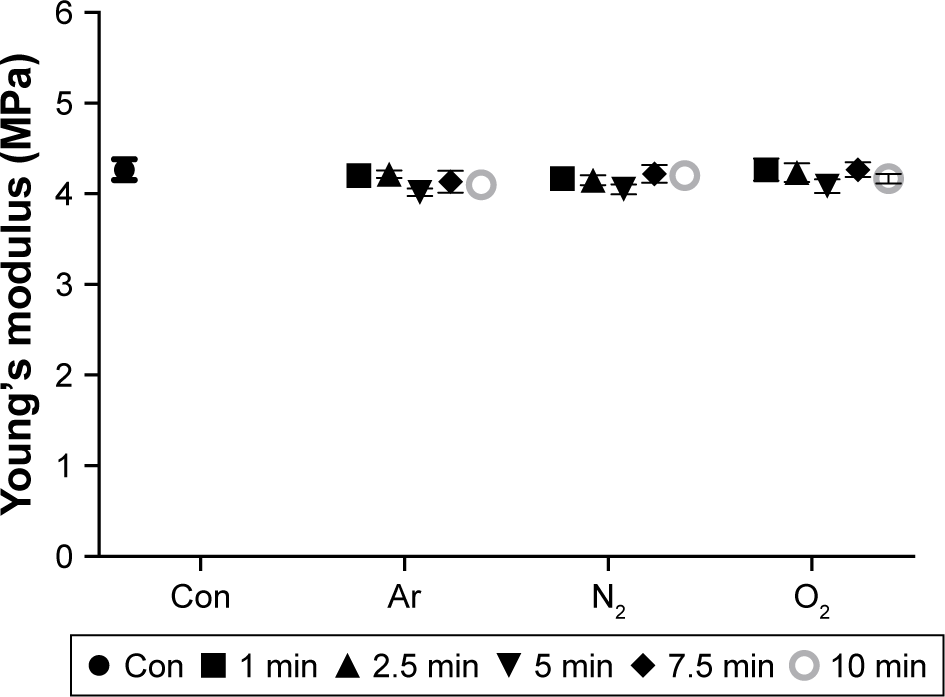

Supplement: Figure S3 — Mechanical properties of the scaffolds after plasma surface modification. Note: Plasma surface modification showed no change in the tensile Young’s elastic modulus for all exposure times (up to 10 minutes). Abbreviations: Ar, argon; Con, untreated; N2, nitrogen; O2, oxygen. [file ijn-13-6123s3.tif]

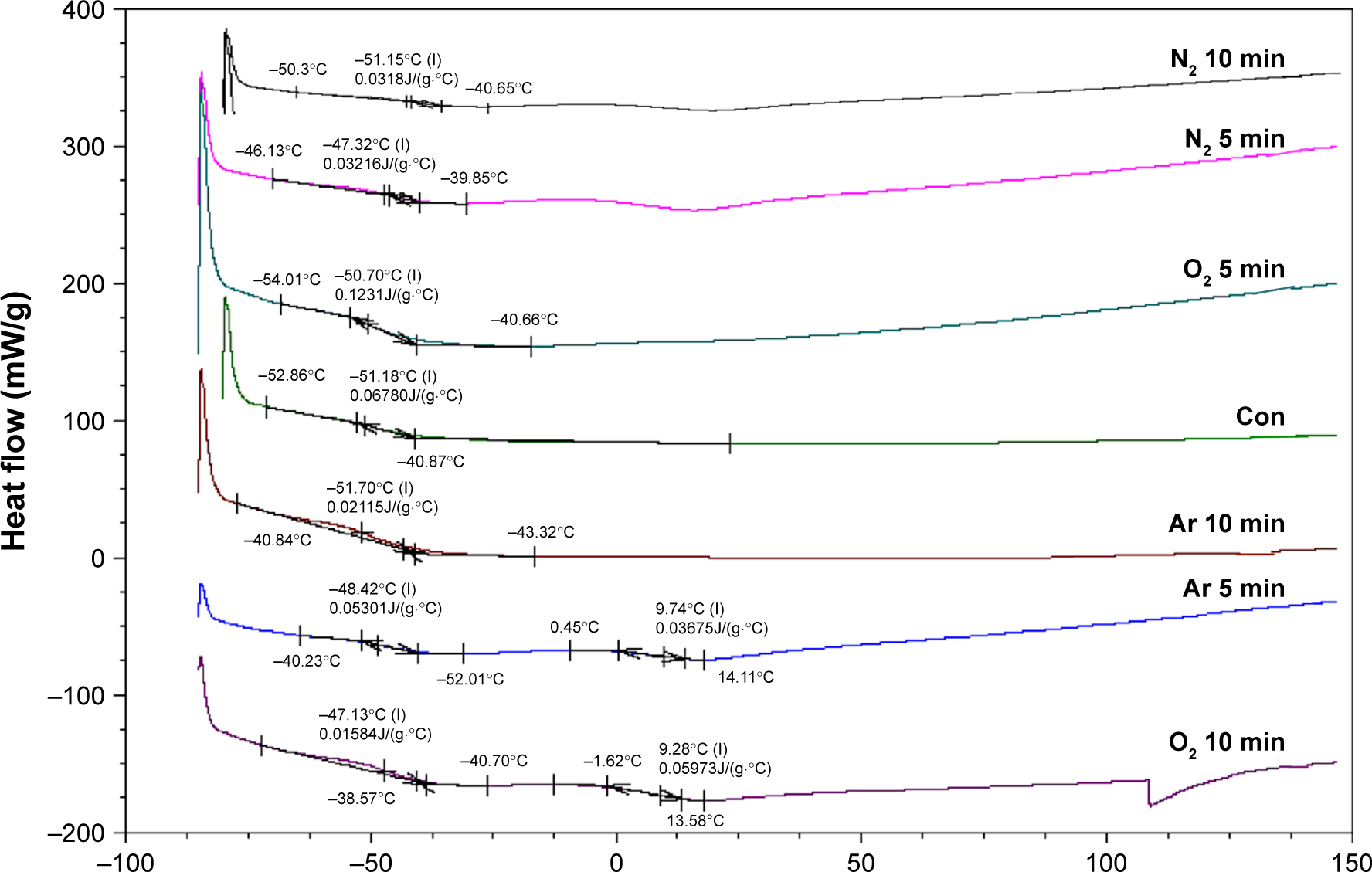

Supplement: Figure S4 — DSC after 5 and 10 minutes of plasma surface modification using Ar, N2 and O2 treatment. Note: This study showed no changes in the Tg after 5 and 10 minutes of plasma surface modification using Ar, N2 or O2 compared to untreated scaffolds (Con). Abbreviations: Ar, argon; DSC, differential scanning calorimetry; N2, nitrogen; O2, oxygen; Tg, glass transition temperature. [file ijn-13-6123s4.tif]

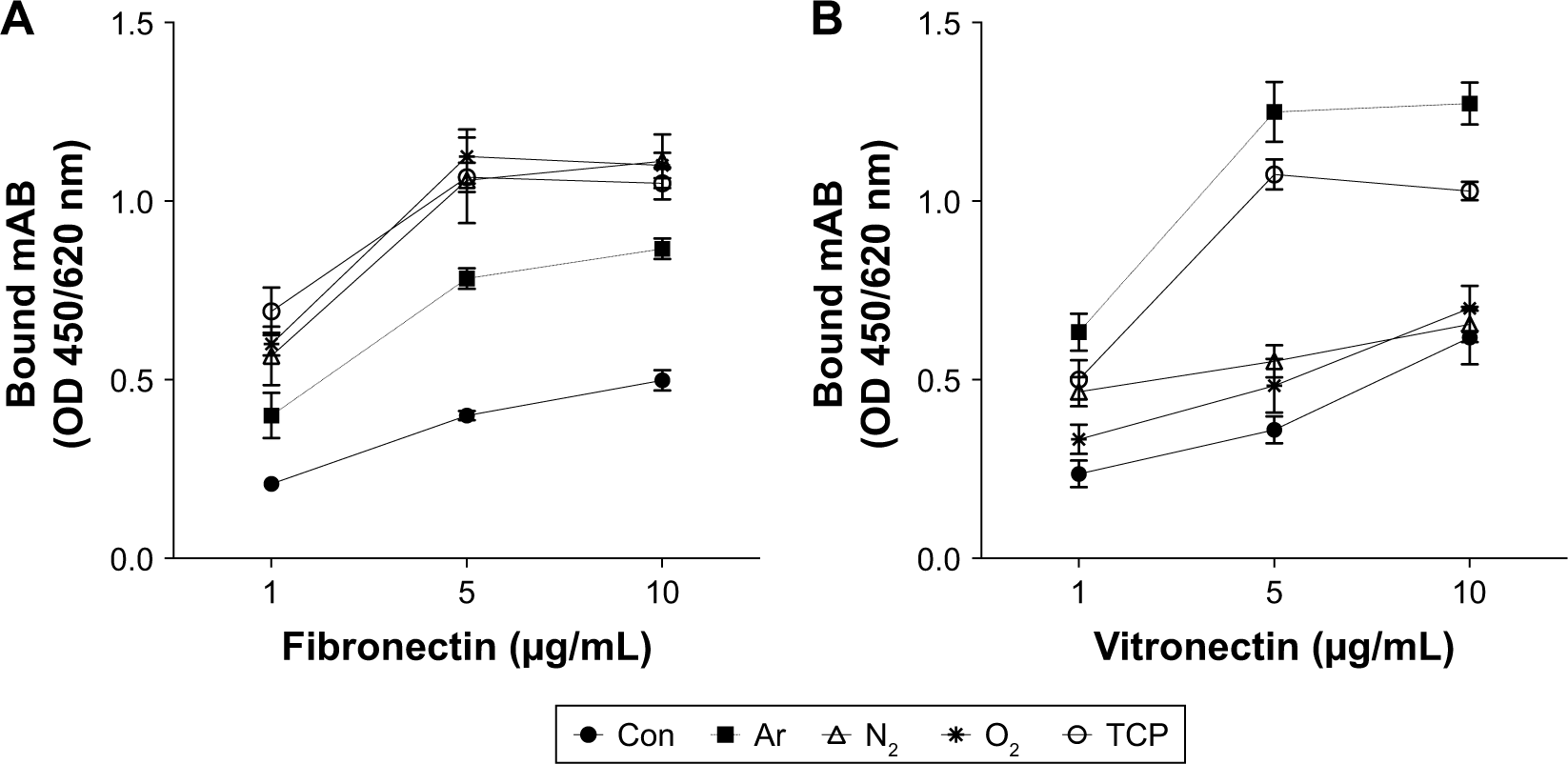

Supplement: Figure S5 — Functional presentation of cell-binding domains of adsorbed fibronectin and vitronectin after plasma surface modification. Total fibronectin (A) and (B) vitronectin protein adsorption onto the scaffolds after 1 hour with 5 minutes of plasma surface modification exposure was analyzed using mAB. Notes: After 5 minutes of N2 or O2 modification, greater amount of fibronectin was absorbed onto the scaffolds compared with Ar-modified and unmodified scaffolds (Con). Vitronectin adsorption was the greatest after 5 minutes of Ar modification compared with all other scaffolds. Abbreviations: Ar, argon; Con, untreated; mAB, monoclonal antibodies; N2, nitrogen; O2, oxygen; TCP, tissue culture plate. [file ijn-13-6123s5.tif]

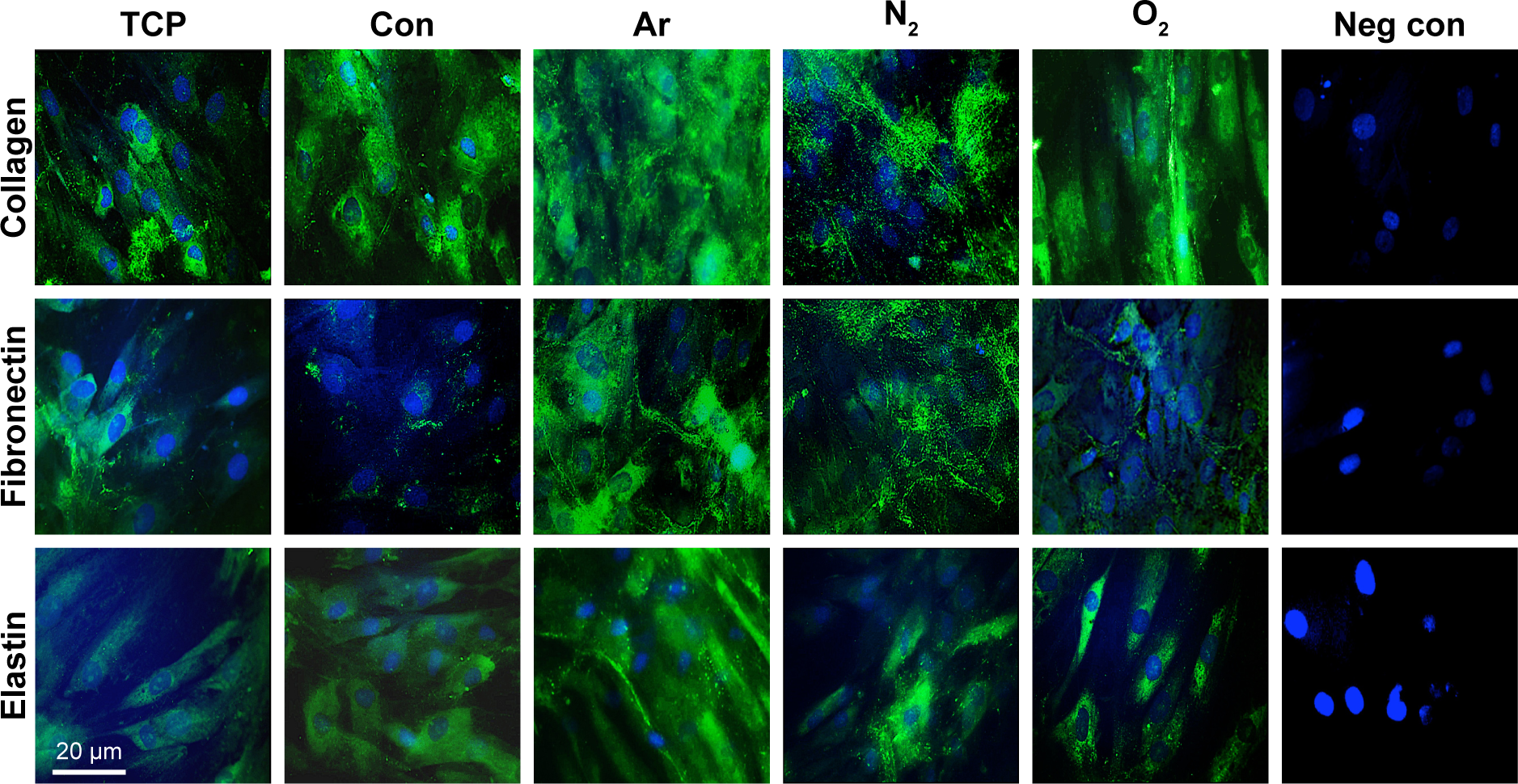

Supplement: Figure S6 — ECM formation by the human dermal fibroblasts after plasma surface modification. Immunocytochemistry confirmed the expression of the ECM markers after 14 days on the plasma-modified and untreated scaffolds (green: collagen type I, elastin, fibronectin; blue: DAPI). Note: Scale bar: 20 µm. Abbreviations: Ar, argon; Con, untreated; ECM, extracellular matrix; N2, nitrogen; Neg con, negative control where primary antibody was omitted; O2, oxygen; TCP, tissue culture plate. [file ijn-13-6123s6.tif]

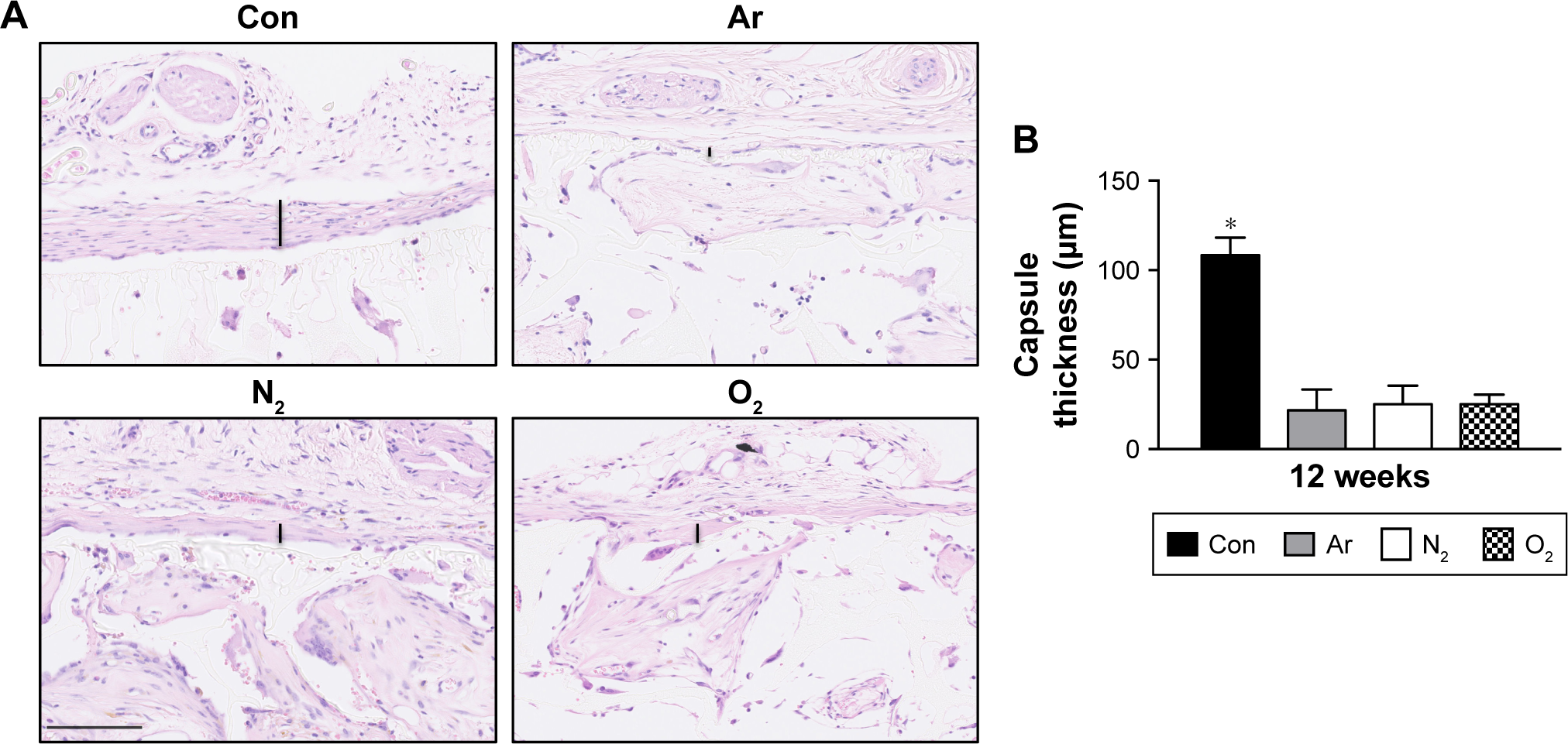

Supplement: Figure S7 — Fibrous capsule formation of the scaffolds treated with plasma surface modification after 12 weeks of subcutaneous implantation. Notes: (A) Histological sections representing thickness of fibrous capsule at the interface of implant and subcutaneous tissue. Scale bar: 200 µm. (B) Quantification of fibrous capsule thickness shows significantly higher fibrous capsule thickness on the unmodified scaffolds compared with plasma-treated scaffold (*P<0.05). Abbreviations: Ar, argon; Con, untreated; N2, nitrogen; O2, oxygen. [file ijn-13-6123s7.tif]

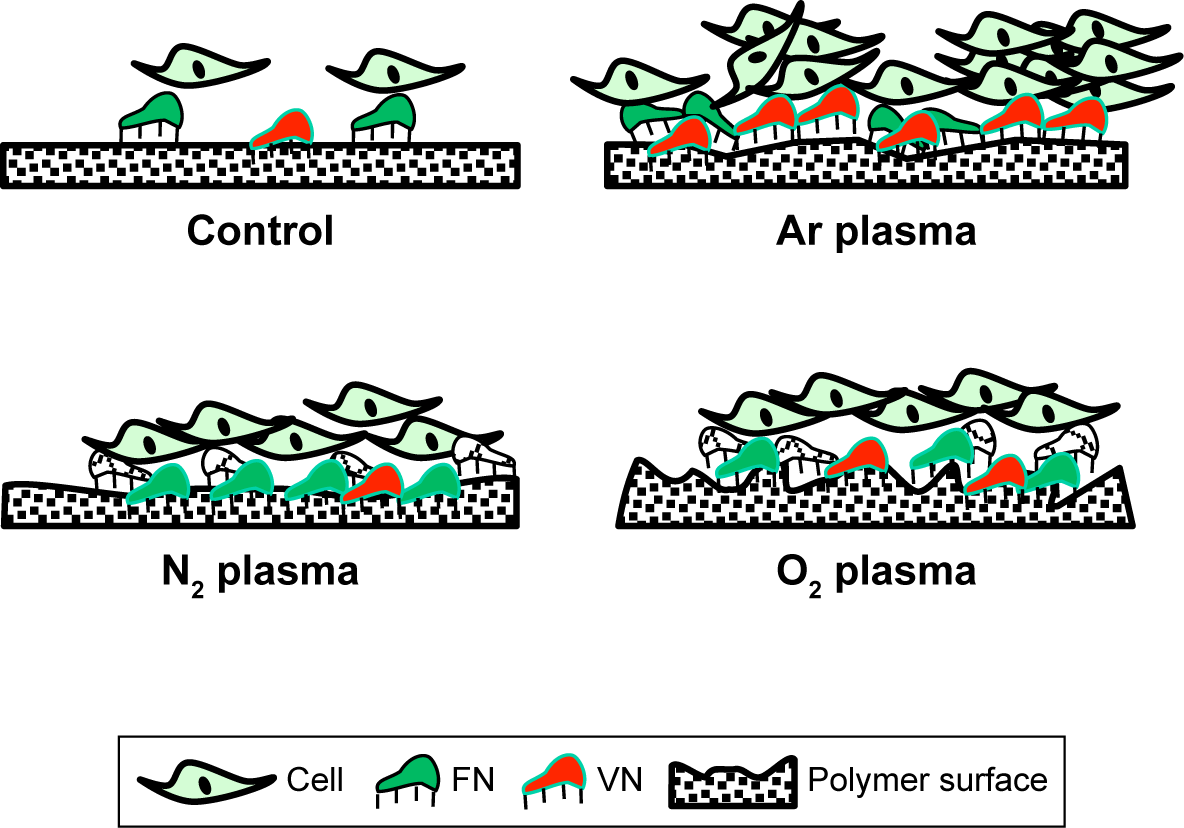

Supplement: Figure S8 — Schematic representation of the mechanism by which cells interact with unmodified and plasma surface-modified scaffolds using Ar, N2 and O2 plasma treatments. Notes: Control scaffolds show lower protein adsorption and cell attachment, Ar-modified scaffolds show moderate roughness, with higher VN adsorption from serum protein, N2 plasma-modified scaffolds show higher FN adsorption compared with O2 plasma-modified scaffolds, which exhibit the highest surface roughness. Image not to the scale. Abbreviations: Ar, argon; FN, fibronectin; N2, nitrogen; O2, oxygen; VN, vitronectin. [file ijn-13-6123s8.tif]
